# Optimized Reduced Field of View and Fat Suppression Methods for Interleaved Multislice In Vivo Cardiac Diffusion Tensor Imaging

**DOI:** 10.1002/mrm.70394

**Published:** 2026-04-22

**Authors:** Yaqing Luo, Pedro F. Ferreira, Ke Wen, Ricardo Wage, Guang Yang, Dudley J. Pennell, Sonia Nielles‐Vallespin, Andrew D. Scott

**Affiliations:** ^1^ National Heart and Lung Institute, Imperial College London London UK; ^2^ Cardiovascular Magnetic Resonance Unit Royal Brompton Hospital, Guy's and St Thomas' NHS Foundation Trust London UK; ^3^ EPSRC Centre for Doctoral Training in Smart Medical Imaging King's College London and Imperial College London London UK; ^4^ Bioengineering Department and Imperial‐X Imperial College London London UK

**Keywords:** cardiac diffusion tensor imaging, fat suppression, in vivo, interleaved multislice imaging, spin echo

## Abstract

**Purpose:**

Slice interleaving, a limited phase encode (PE) field of view (FOV), and effective fat suppression are vital for efficient cardiac diffusion tensor imaging (cDTI) with minimal artifacts. This study aimed to optimize reduced FOV and fat suppression methods for interleaved multislice cDTI to improve signal‐to‐noise ratio (SNR) and minimize artifacts.

**Methods:**

Two‐slice motion compensated spin echo datasets from 20 healthy volunteers were acquired. Four reduced PE FOV sequences were evaluated: 2DRF pulse; applying either $180^{{}^{\circ}}$ or $90^{{}^{\circ}}$ pulses in PE direction; and the proposed flip‐back sequence with a nonselective $180^{{}^{\circ}}$ pulse after readout to restore inverted magnetization. Four fat suppression techniques were implemented: no fat suppression (standard); fat saturation; binomial water excitation and spectral attenuated inversion recovery (SPAIR).

**Results:**

The proposed flip‐back sequence with SPAIR achieved the highest median SNR, and its SNR values are significantly higher (p<0.01) than 2DRF with SPAIR as current state‐of‐the‐art. SPAIR and water excitation demonstrated comparable performance when combined with the flip‐back sequence, and both yielded superior image quality than with no suppression or fat saturation. SPAIR showed robust fat suppression across most subjects, whilst water excitation exhibited advantages in some subjects with a high body mass index.

**Conclusion:**

The proposed flip‐back sequence with SPAIR enables efficient interleaved multislice imaging with reduced PE FOV and effective fat suppression, facilitating clinical translation of in vivo cDTI.

## 1 Introduction

Cardiac diffusion tensor imaging (cDTI) reveals valuable insights on in vivo myocardial microstructure in a noninvasive manner [1, 2, 3, 4]. It provides useful information on cardiovascular diseases [5], including dilated [3, 6] and hypertrophic cardiomyopathies [3, 7, 8, 9], and myocardial infarction [10, 11, 12]. However, it is typically a low signal‐to‐noise ratio (SNR) [13] technique, which introduces bias and uncertainty into the quantitative parameters extracted from the images [14, 15].

While stimulated echo acquisition mode (STEAM) sequences are robust to motion throughout the cardiac cycle [16], velocity and acceleration compensated or second‐order motion compensated spin echo (MC‐SE) cDTI sequences are desirable for SNR efficiency. They have short diffusion times, allowing for the acquisition of each image within a single cardiac cycle, and the inherent 50% signal loss associated with stimulated echoes can be avoided [17].

Recent developments in ultra‐high performance gradient systems allow for further improvements in the performance of MC‐SE cDTI sequences [18, 19]. These enhancements enable shorter diffusion gradient durations, resulting in reduced echo times and a higher SNR. Additionally, shorter gradient durations may contribute to reduced sensitivity to motion.

Acquisition of cDTI is typically performed using a single‐shot echo planar imaging (EPI) readout [1, 20, 21], which enables rapid data acquisition. However, EPI is highly sensitive to changes in magnetic susceptibility, eddy currents and off‐resonance effects, leading to geometric distortions, signal loss, and blurring, particularly as readout duration increases [16, 22, 23]. These artifacts are exacerbated by the long echo train and low bandwidth per pixel in the phase encoding (PE) direction [24, 25]. To mitigate these EPI‐related effects, a reduced PE field of view (FOV) is often employed, which enables a shortened EPI readout, and therefore reduces artifacts [26]. In the case of spin echo, this is often achieved by making the $90^{{}^{\circ}}$ excitation pulse slice‐selective in the PE direction, restricting the tissue generating signal to a narrower region and preventing aliasing artifacts from surrounding tissues [21, 27]. A spatially‐selective $180^{{}^{\circ}}$ refocusing pulse is then applied orthogonally in the slice direction for inner volume imaging [15]. Two‐dimensional spatially‐selective RF (2DRF) pulses offer an alternative approach [28, 29]. Unlike conventional one‐dimensional slice‐selective pulses, 2DRF pulses excite a two‐dimensional region. When designed to be selective in both the slice and phase encoding directions, 2DRF excitation restricts the excited magnetization to a well‐defined inner volume, thereby reducing the FOV [30]. This targeted excitation allows a shorter EPI readout train, avoiding aliasing from anatomy outside the imaged FOV [31] at the expense of a longer RF pulse. These reduced PE FOV strategies improve image quality while preserving the advantages of EPI‐based cDTI acquisitions.

Interleaved multislice acquisitions enhance SNR efficiency [28] by increasing the effective TR per slice. However, slice interleaving is more challenging when the FOV is reduced by applying the excitation pulse in the PE direction due to saturation of magnetization outside the imaged slice.

A further challenge in MC‐SE cDTI arises from the multiple sources of fat neighboring the myocardium (e.g., epicardial, pericardial, chest wall and back), frequently causing fat artifacts [32]. Due to the chemical shift between fat and water, fat signals are spatially shifted by the EPI readout and can often be superimposed on the myocardium, deteriorating image quality and leading to errors in diffusion tensor quantification. Chemical shift selective based fat saturation, is often used in cDTI to reduce fat signals. However, the performance of fat saturation can be affected by $B_{0}$ and $B_{1}$ inhomogeneities, which reduce frequency selectivity and can lead to incomplete fat suppression and signal loss in the myocardium [33].

This work aims to optimize reduced FOV and fat suppression techniques for interleaved multislice MC‐SE cDTI. We propose reducing PE FOV by applying the $180^{{}^{\circ}}$ refocusing pulse along the PE direction [34, 35], followed by an additional nonselective $180^{{}^{\circ}}$ pulse immediately after the readout, which restores the inverted magnetization outside the imaged slice for compatibility with interleaved multislice imaging. We also compare cDTI parameters and data quality across different fat suppression techniques in combination with the proposed flip‐back sequence, and compare our optimized pairing with a current state‐of‐the‐art method.

## 2 Methods

### 2.1 Reduced PE FOV Sequences

**FIGURE 1:**
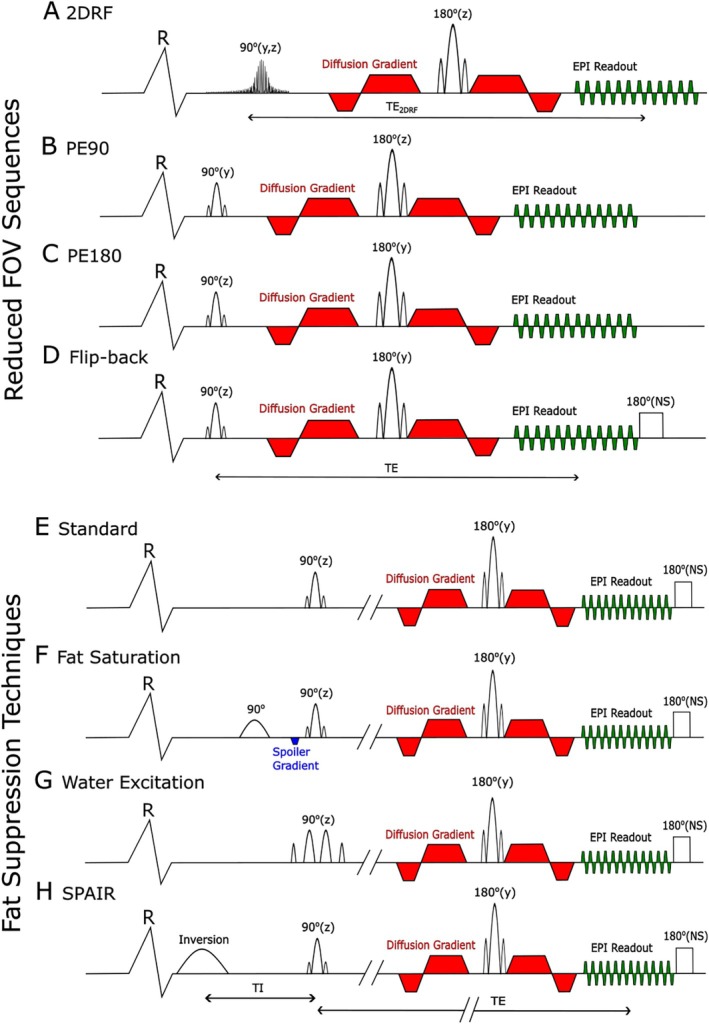
Sequence schematics for four reduced PE FOV sequences including (A) 2DRF, (B) $90^{{}^{\circ}}$ RF pulse in the PE direction (PE90), (C) $180^{{}^{\circ}}$ RF pulse in the PE direction (PE180), and (D) the proposed flip‐back sequence which has an additional nonselective $180^{{}^{\circ}}$ pulse after readout, and four fat suppression techniques with the proposed flip‐back sequence including (E) no suppression (Standard), (F) fat saturation, (G) binomial water excitation, and (H) spectral attenuated inversion recovery (SPAIR).

Four approaches for reducing the FOV along the PE direction were implemented for in vivo MC‐SE EPI cDTI (Figure 1) [36, 37], and combined with SPAIR as described in Section 2.2:

- The 2DRF sequence used a $90^{{}^{\circ}}$ spatially‐selective pulse to limit the excitation in both the slice selective (SS) and PE directions (ZoomIt) [28], followed by a $180^{{}^{\circ}}$ pulse in the SS direction (Figure 1A).
- The PE90 sequence applied the $90^{{}^{\circ}}$ RF pulse in the PE direction, and $180^{{}^{\circ}}$ RF pulse in the SS direction [16] to image a zonal region (Figure 1B).
- As an alternative, the PE180 sequence applied the $90^{{}^{\circ}}$ pulse in the SS direction and $180^{{}^{\circ}}$ pulse in the PE direction, to image the same reduced region of interest as the PE90 sequence (Figure 1C).
- The proposed flip‐back sequence modified the PE180 sequence with an additional nonselective flip‐back $180^{{}^{\circ}}$ pulse immediately after the EPI readout (Figure 1D). Within the imaged slice, the additional non‐selective $180^{{}^{\circ}}$ pulse leaves the magnetization in a relatively similar state. Outside the imaged slice, the first $180^{{}^{\circ}}$ pulse inverts the magnetization and the second $180^{{}^{\circ}}$ pulse flips it back to its original state.

The difference between the four reduced FOV sequences is in the excitation and refocusing RF pulses, thus affecting the longitudinal magnetization $M_{z}$ for each slice in interleaved multislice imaging. Figure S1 provides a schematic representation of the behavior of $M_{z}$ for the reduced FOV sequences for two‐slice acquisition. The role of the additional flip‐back pulse in the proposed sequence is to ensure $M_{z}$ in the slice not currently being imaged is relatively unaffected before the $90^{{}^{\circ}}$ excitation, in order to maximize SNR. It is worth noting that the flip‐back pulse is not suitable for PE90, because the level of $M_{z}$ that can be recovered is small.

A key distinction in our proposed flip‐back sequence compared to previous use of the flip‐back pulse in the literature [34, 35, 38, 39], is the use of a nonselective, rather than slice selective, flip‐back pulse. The two approaches were initially evaluated in an agar phantom ($T_{1}=1270\;\mathrm{ms}$, $T_{2}=38\;\mathrm{ms}$, diameter ≈196mm) using the flip‐back sequence described above and the protocol used for the in vivo scans.

### 2.2 Fat Suppression Techniques

Four fat suppression methods were compared when combined with the proposed flip‐back sequence, in order to minimize fat artifacts in MC‐SE EPI cDTI. All techniques were based on the default vendor supplied implementations. These are respectively:

1. No fat suppression or “standard” (Figure 1E).
2. Fat saturation via chemical shift selective saturation and spoiling (Figure 1F), implemented a frequency‐selective RF pulse tuned to the fat frequency, followed by a spoiling gradient to dephase the excited fat signal, effectively suppressing it [40].
3. Binomial water selective excitation with a 4 pulse design (Figure 1G), used multiple RF pulses with a binomial series weighting of the flip angles (i.e., $11^{{}^{\circ}}-34^{{}^{\circ}}-34^{{}^{\circ}}-11^{{}^{\circ}}$) and timing spaced to allow for $180^{{}^{\circ}}$ phase shift between fat and water signals to selectively excite only water while minimizing fat excitation [41, 42].
4. SPAIR (Figure 1H), applied an inversion pulse that selectively targeted fat, inverting its magnetization without affecting water. The excitation for imaging was applied at the inversion time (TI) when the fat signal was nulled [43, 44, 45].

It is worth noting that 2DRF is incompatible with water excitation.

### 2.3 Study Design

We compared the four methods of reduced FOV and four fat suppression techniques when used with the proposed flip‐back sequence.

#### 2.3.1 Data Acquisition

Short‐axis breath hold cDTI data using interleaved multislice MC‐SE EPI were acquired from 20 healthy volunteers, including 15 females and 5 males, with a mean age of 29 years and range of 21–44 years, and BMI ranges from 19.3 to 28.5 $\mathrm{kg}/m^{2}$. The acquisition was triggered to peak systole at 3 T (Cima.X Siemens Healthcare, Forchheim, Germany) with a maximal gradient strength and slew rate of 200mT/m and 200 T/m/s respectively. An 18‐element anterior coil and 8–12 elements of a posterior spine coil were used. The study was conducted in accordance with National Research Ethics Service approval. Trigger delay timings were determined from systolic time points from two‐slice short axis CINE acquisition in the same planes as the cDTI images, and adjusted according to reduced FOV and fat suppression techniques to ensure that central *k*‐space data was acquired at peak systole. SPAIR was implemented with a fixed TI of 182 ms, based on the assumed RR‐interval of 1000 ms [46].

Spatial resolution was $2.8\times 2.8\times 8\;{\mathrm{mm}}^{3}$, and FOV was $135\times 360\;{\mathrm{mm}}^{2}$. For each sequence, eight breath holds were acquired consisting of one $b=0\;s/{\mathrm{mm}}^{2}$ image and six diffusion encoding directions at $b=450\;s/{\mathrm{mm}}^{2}$, and two breath holds with $b=0\;s/{\mathrm{mm}}^{2}$ data and six diffusion encoding directions at $b=50\;s/{\mathrm{mm}}^{2}$. Interleaved two‐slice imaging with 8mm inter‐slice gap was performed, resulting in TR = 2RR‐intervals. For all breath holds, the first four cardiac cycles were used to acquire EPI phase correction data and parallel imaging reference data in the two slices. This resulted in an 18 RR‐interval breath hold. All sequences have TE=50ms, apart from the 2DRF sequence which has TE=65ms, due to the much longer duration of the RF pulse from the peak to end. Factor 2 parallel imaging (SENSE) was enabled, and data were reconstructed online using the standard product reconstruction with a final reconstructed pixel size of $1.4\times 1.4\;{\mathrm{mm}}^{2}$ (reduced from $2.8\times 2.8\;{\mathrm{mm}}^{2}$ acquired resolution using zero filling in *k*‐space). Partial Fourier sampling was not used.

#### 2.3.2 Analysis

Post‐processing was performed using the open‐source Python pipeline “INDI” developed by our group https://github.com/ImperialCollegeLondon/INDI [47, 48]. The pixel‐wise diffusion tensor was calculated using a nonlinear least‐squares algorithm with $b=450\;s/{\mathrm{mm}}^{2}$ and $b=50\;s/{\mathrm{mm}}^{2}$ images, excluding $b=0\;s/{\mathrm{mm}}^{2}$ images to minimize the contribution of blood signal to the cDTI measures.

Sequences were compared using measures of median left ventricular (LV) fractional anisotropy (FA), mean diffusivity (MD), absolute sheetlet angle (E2A), helix angle (HA), and transmural helix angle gradient (HAG), SNR, proportion of negative eigenvalues in the LV and transverse angle (TA). Papillary muscles and the septo‐marginal trabeculations of the right ventricle were excluded from the LV region of interest used in quantification. SNR in the LV region was measured for each diffusion direction at $b=450\;s/{\mathrm{mm}}^{2}$ using the multiple repetitions technique [21]. Median LV SNR was extracted and averaged across directions. HAG in units of 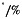 myocardial thickness was calculated by linear regression of transmural HA line profiles from endocardium to epicardium to quantify HA.

Quality indicators for cDTI included the percentage of negative eigenvalues in the LV, the linearity of the HAG fit using $R^{2}$ [1, 17, 49], absolute value of HAG which should be close to 1 [16, 50], and the standard deviation of TA which should be small in healthy myocardium.

Subjective assessment of cDTI data quality was performed by scoring the HA maps. A circumferential linear variation of HA from epicardium to endocardium was assumed. Consistent with prior studies [16, 50], HA map scoring was based on a score of 3 for HA maps with visually >95% normal pattern on transmural HA variation, 2 for >75%, 1 for >50% and 0 for <50%. The severity of the fat artifacts was scored collectively on average magnitude images, FA and MD maps to compare between fat suppression techniques. A score of 3 was given to results with fat artifact appearance in <5% area of the LV, 2 for <25%, 1 for <50% and 0 for >50%. Scoring was performed blinded in a randomized order.

A Wilcoxon signed‐rank test was performed for nonparametric statistical analysis, calculated *p*‐values were corrected using the Benjamini–Hochberg (BH) method for multiple comparisons [51] and p<0.05 was considered significant. For comparison between reduced FOV sequences, the proposed flip‐back sequence was the reference method. For fat suppression techniques, flip‐back with SPAIR was the reference that other methods were compared to.

## 3 Results

### 3.1 Reduced PE FOV

The phantom study demonstrated that the nonselective flip‐back pulse (mean ± standard deviation, 54.2 ± 11.8) results in a higher SNR than the PE‐selective flip‐back pulse (49.0 ± 12.5), which is more uniform across the phantom. Full details of the acquisition and SNR maps are provided in Figure S2.

**FIGURE 2:**
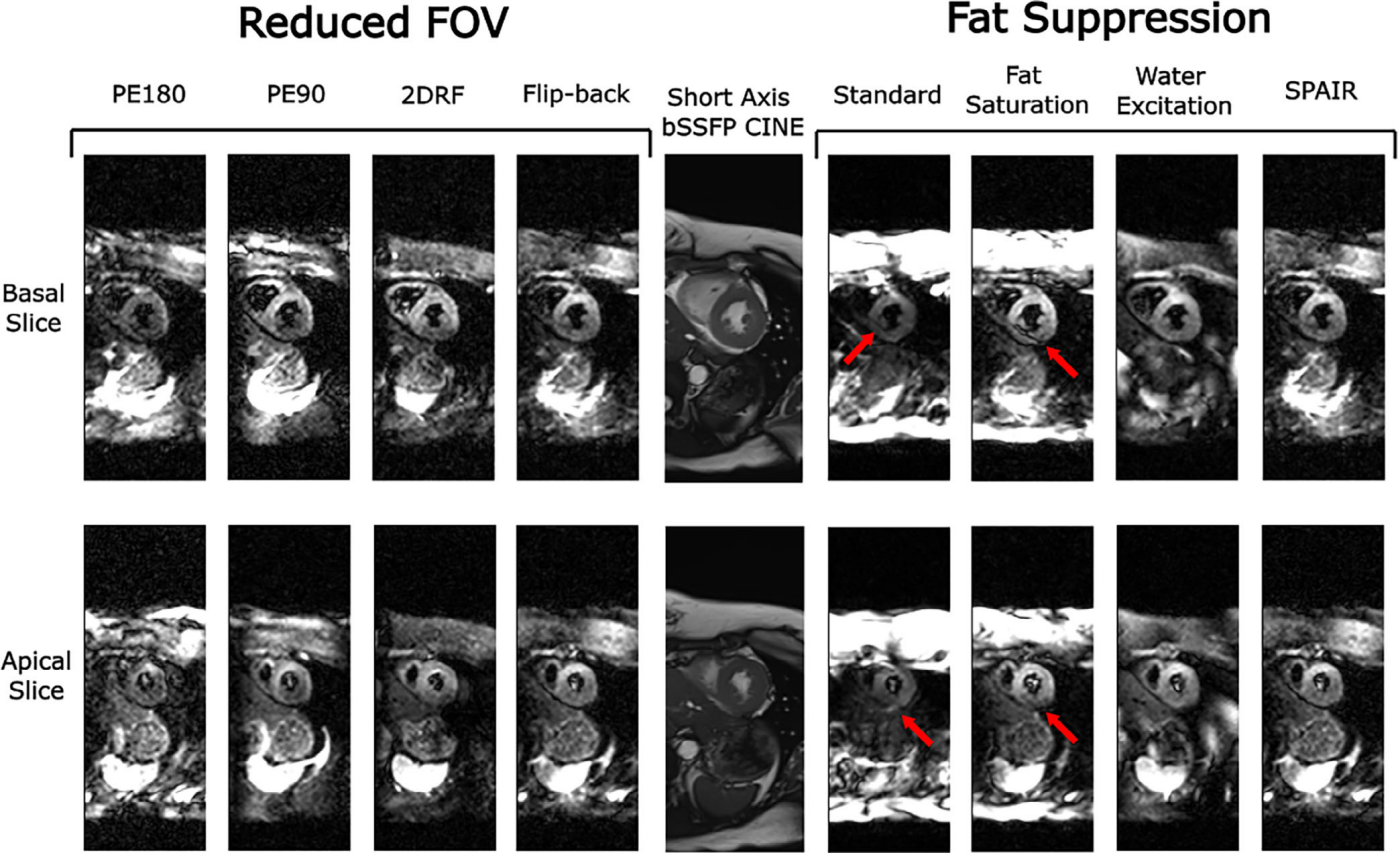
Example $b=450\;s/{\mathrm{mm}}^{2}$ magnitude cDTI images from the different reduced FOV sequences and flip‐back sequence with different fat suppression methods. Peak systolic frames of bSSFP cine images acquired in the same plane are shown for reference. Red arrows highlight artifacts. Results from the flip‐back sequence with both water excitation and SPAIR demonstrate excellent visual image quality with no fat artifacts in the myocardium.

**FIGURE 3:**
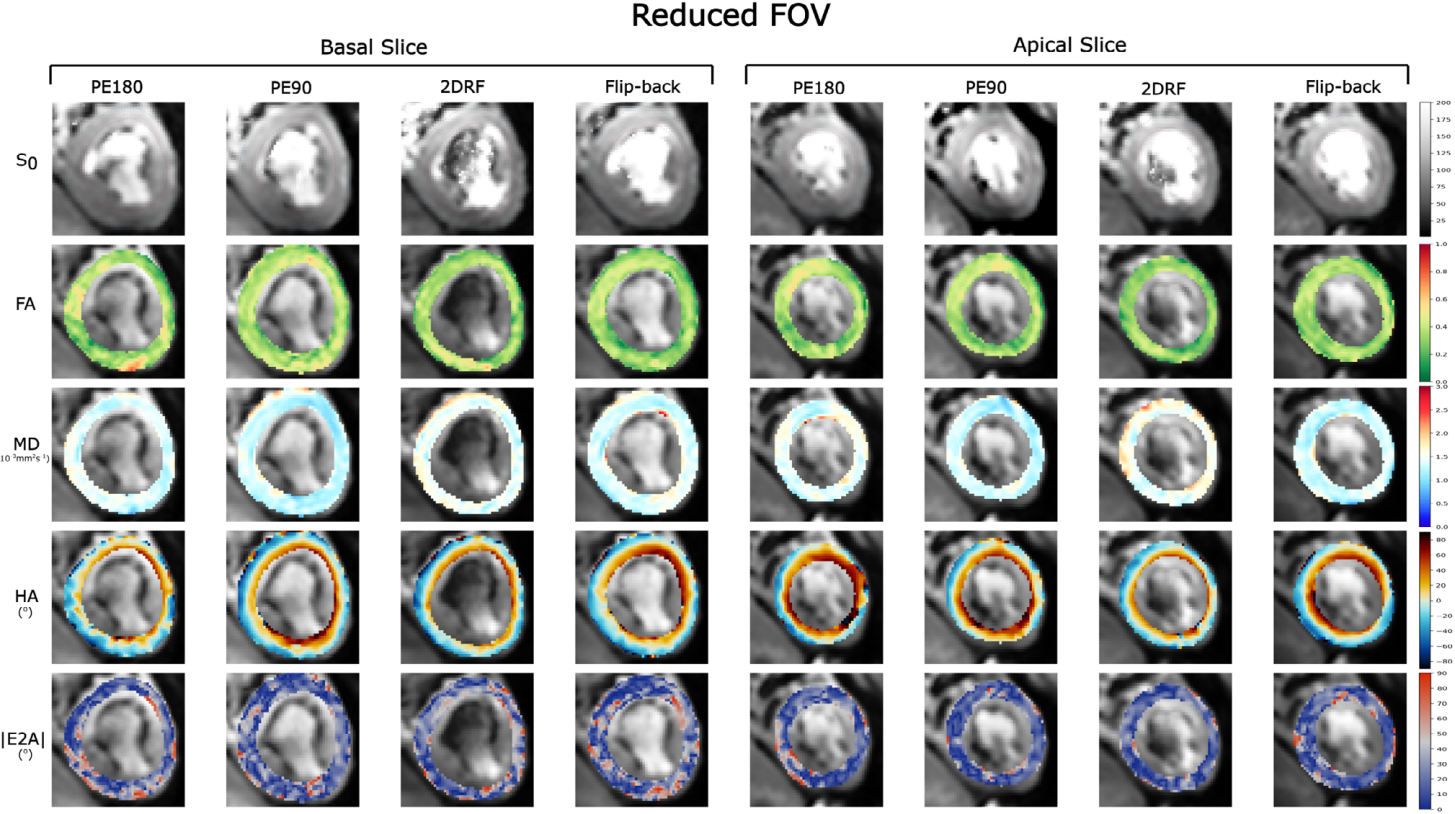
Comparison of example cDTI maps including average magnitude image, FA, MD, HA and absolute E2A, at basal and apical slices for reduced FOV sequences including PE180, PE90, 2DRF, and proposed flip‐back sequence.

Example $b=450\;s/{\mathrm{mm}}^{2}$ magnitude cDTI images for basal and apical slices for the four reduced FOV methods are shown in Figure 2. The images demonstrate a visually apparent decrease in SNR for the PE180 method. Examples of cDTI parameter maps are illustrated in Figure 3.

**FIGURE 4:**
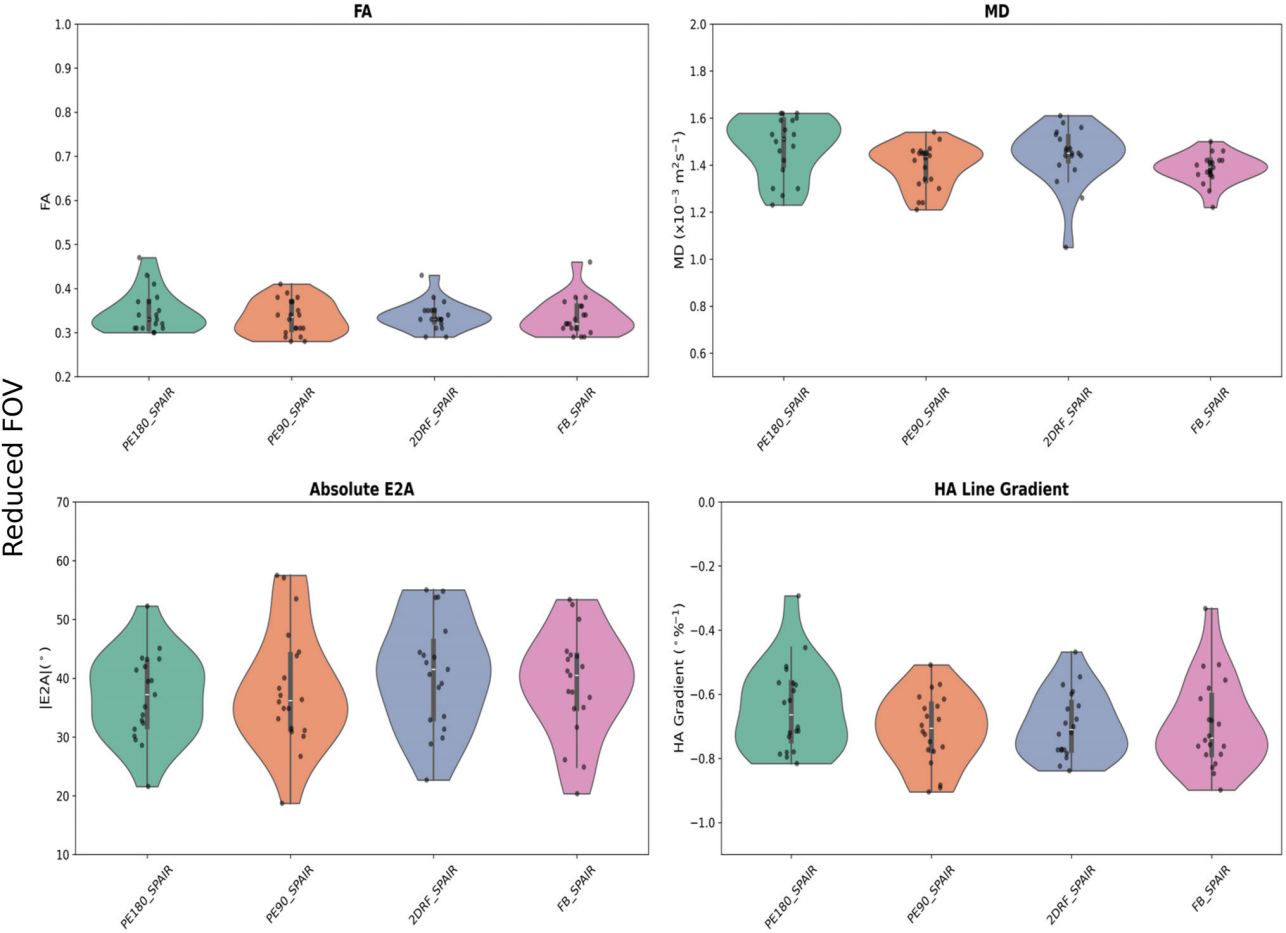
Comparison of cDTI parameters across reduced FOV sequences including PE180, PE90, 2DRF, and flip‐back. Violin plots of cDTI parameter metrics including FA, MD, absolute E2A and transmural HA gradient. The median is shown as a white line in each plot, together with the interquartile range.

**FIGURE 5:**
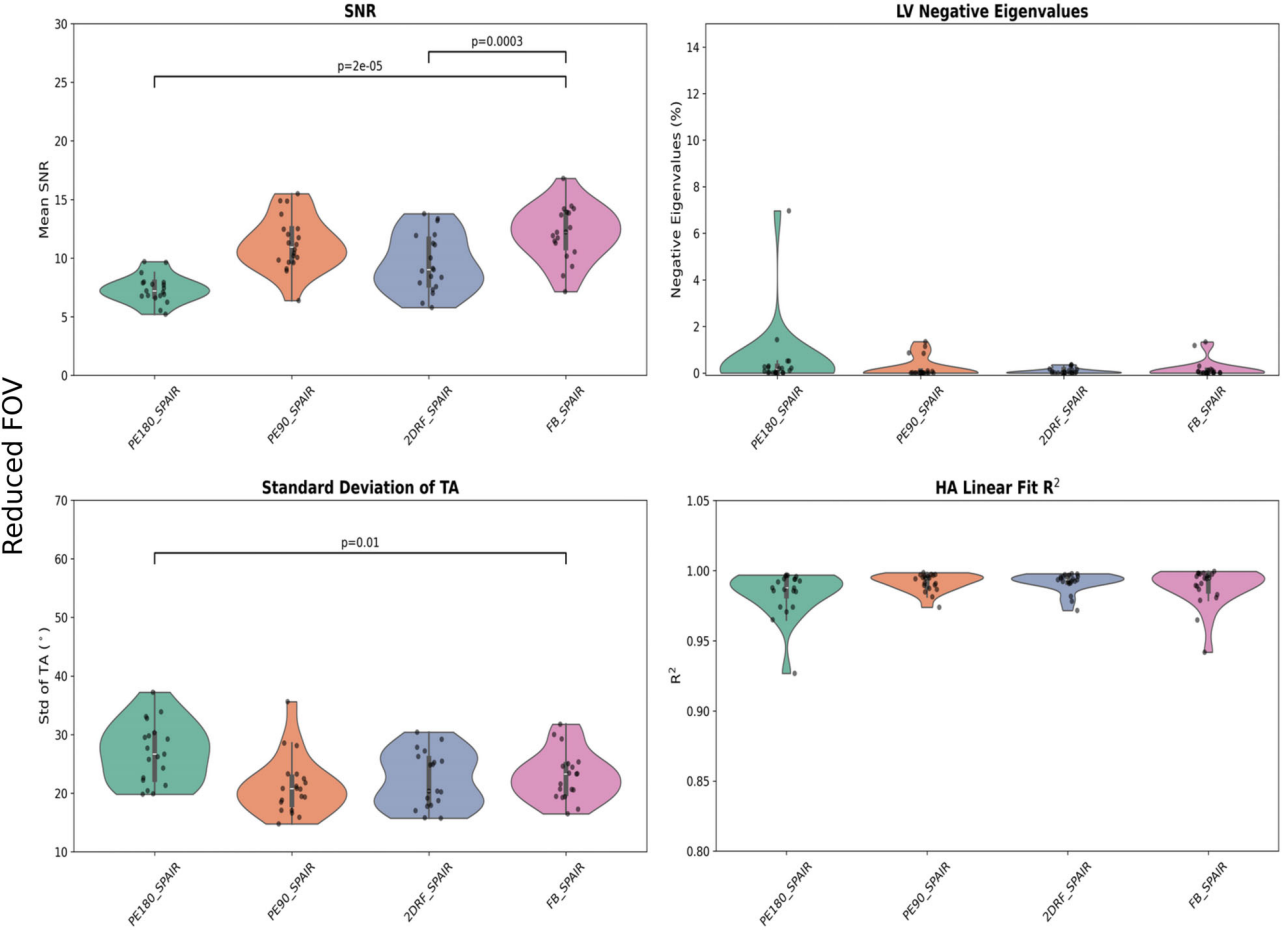
Comparison of cDTI quality metrics across reduced FOV sequences including PE180, PE90, 2DRF, and flip‐back. Distributional violin plots for cDTI quality metrics including SNR, percentage of negative eigenvalues in the LV, the standard deviation of TA and $R^{2}$ of HA linear fit.

cDTI parameters, FA, MD, E2A, and HAG were generally similar between reduced FOV techniques, as shown in Figure 4. No significance was observed across these parameters. As demonstrated by Figure 5, comparing cDTI measures of data quality, the PE180 sequence demonstrated the lowest acquisition quality (quoted as elsewhere as median[interquartile range]). It has lower SNR values (7.2[1.1], p<0.01) and higher standard deviation of TA (26.7[7.6]°, p=0.01) than flip‐back (12.2[3.0] and 23.3[4.8]°, respectively), suggesting lower data quality.

Although MD values have no significant differences between the reduced FOV sequences, it can be observed that the flip‐back sequence (1.39[0.06]$\times 10^{-3}{\mathrm{mm}}^{2}s^{-1}$) has a more tightly packed distribution of values and smaller interquartile range than the 2DRF sequence (1.45[0.10]$\times 10^{-3}{\mathrm{mm}}^{2}s^{-1}$).

Of all the reduced FOV techniques, flip‐back (12.2[3.0]) has the highest SNR amongst the $b=450\;s/{\mathrm{mm}}^{2}$ images, in particular, its SNR is significantly higher than the 2DRF sequence (9.0[3.9], p<0.01), as demonstrated in Figure 5. Median SNR for flip‐back is 12% and 36% higher than PE90 and 2DRF respectively.

Qualitative assessment of HA map quality was performed on reduced FOV sequences, and results are provided in Figure S3. Flip‐back (2.0[0.8]) scored highest overall and significantly higher than PE180 (1.0[1.3], p<0.01).

### 3.2 Fat Suppression

Performance across four different fat suppression methods with flip‐back sequence were evaluated. Figure 2 shows example two‐slice $b=450\;s/{\mathrm{mm}}^{2}$ magnitude cDTI images for flip‐back sequence with no fat suppression, fat saturation, water excitation and SPAIR, respectively. For both water excitation and SPAIR, the artifacts are negligible. Figure S4 demonstrates that images acquired using flip‐back with water excitation and with SPAIR maintain the best image quality, across the subjects with low BMI, high BMI, epicardial fat and visceral fat. In particular, images acquired using fat saturation include fat artifacts superimposed onto the myocardium for subjects with high BMI, epicardial fat and visceral fat, and are comparable to water excitation and SPAIR when subject has low BMI.

**FIGURE 6:**
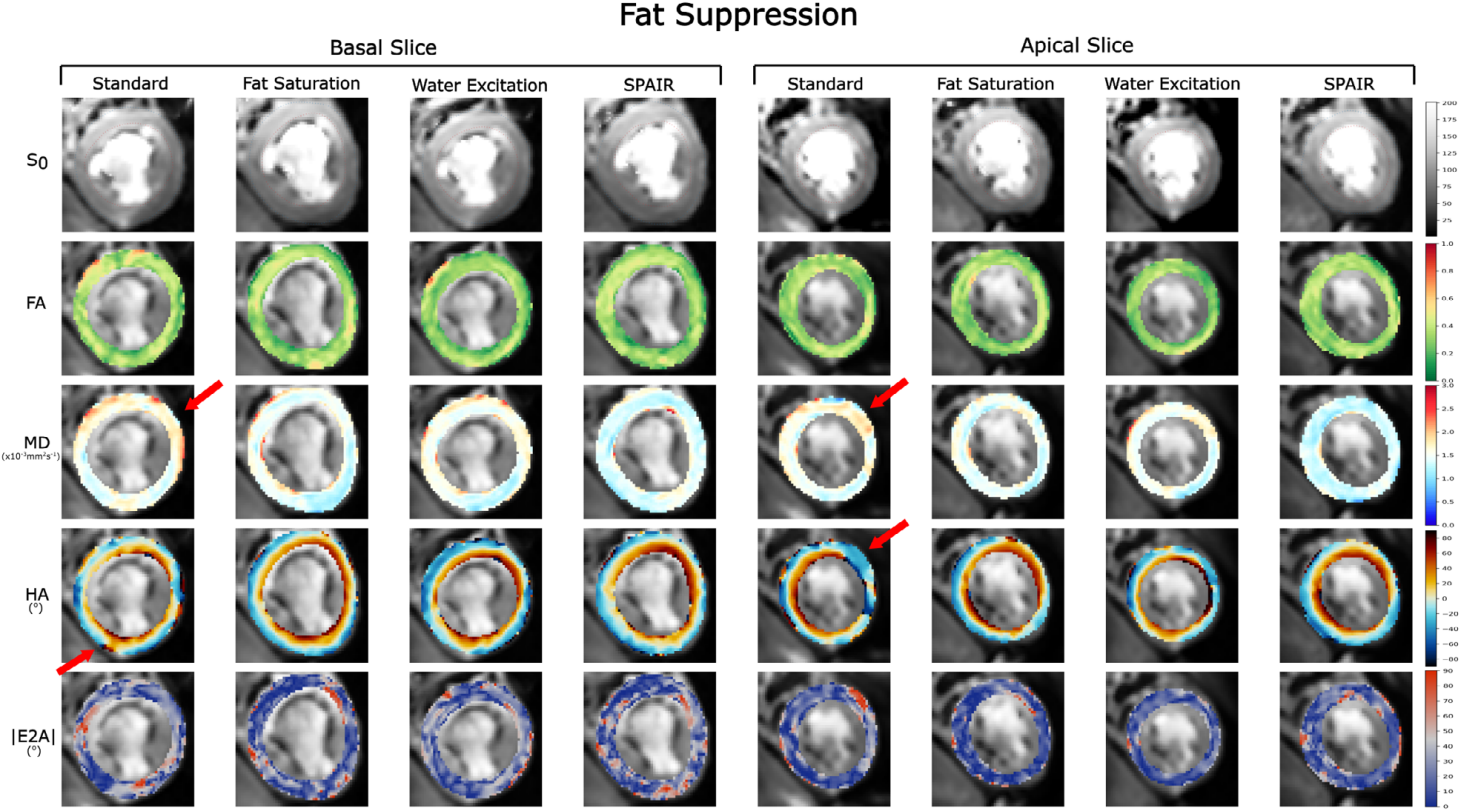
Comparison of example cDTI maps including average magnitude image, FA, MD, HA and absolute E2A, at basal and apical slices for fat suppression techniques with the flip‐back sequence including no suppression (standard), fat saturation, water excitation and SPAIR. Red arrows highlight the regions affected by fat artifacts. Results from the flip‐back sequence with both water excitation and SPAIR visually demonstrate the best quality.

Examples of cDTI parameter maps are illustrated in Figure 6. Example maps of a subject with high BMI are given in Figure S5. It is worth noticing that images with no suppression fail in this case, images with fat saturation are severely affected by fat artifacts, and SPAIR was not able to effectively suppress fat artifacts. Images with water excitation are the least affected by fat artifacts in this comparison.

**FIGURE 7:**
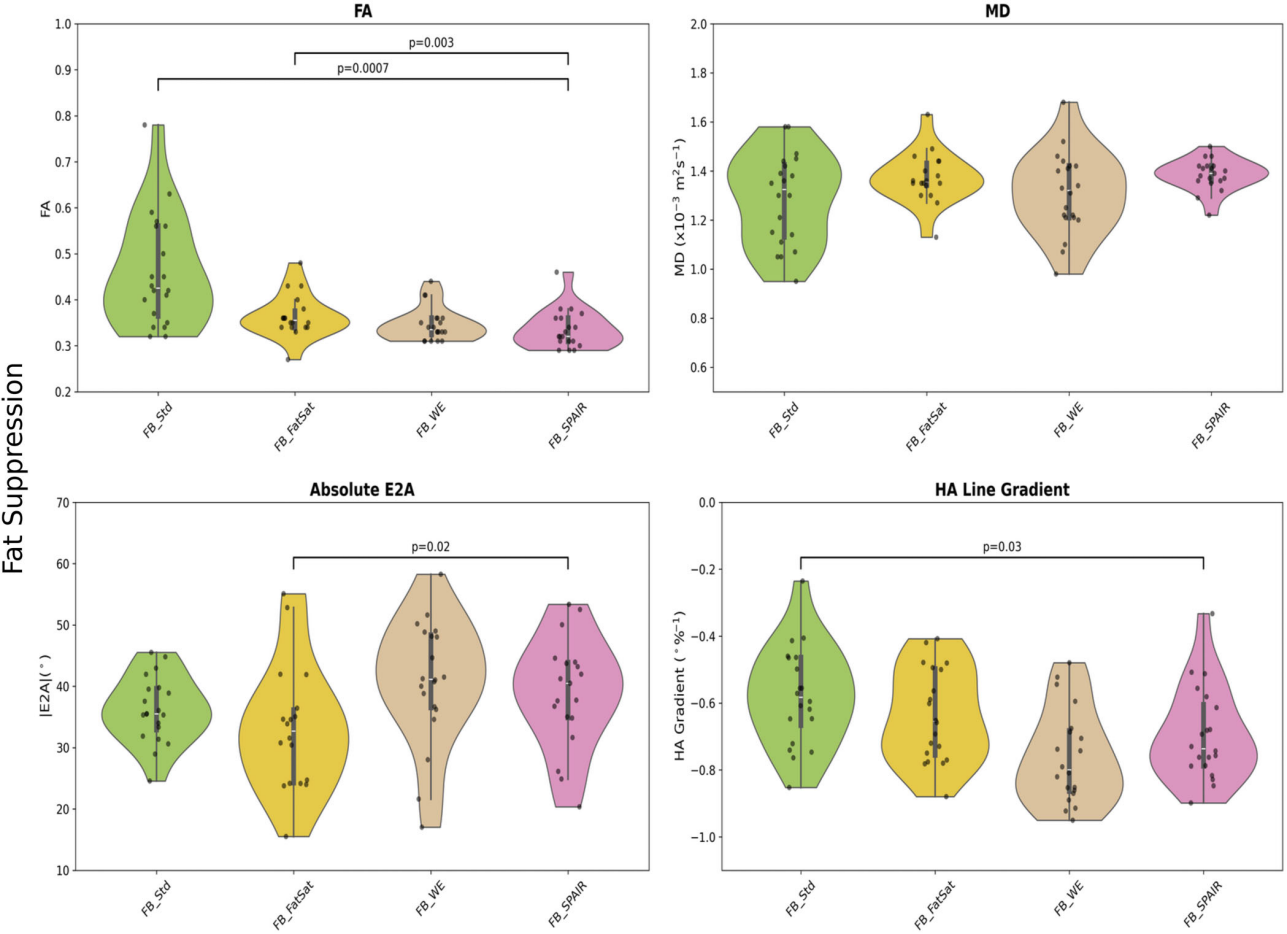
Comparison of cDTI parameters across fat suppression techniques including flip‐back with no suppression (standard), fat saturation, water excitation and SPAIR. Violin plots of cDTI parameter metrics including FA, MD, absolute E2A and transmural HA gradient. The median is shown as a white line in each plot, together with the interquartile range.

**FIGURE 8:**
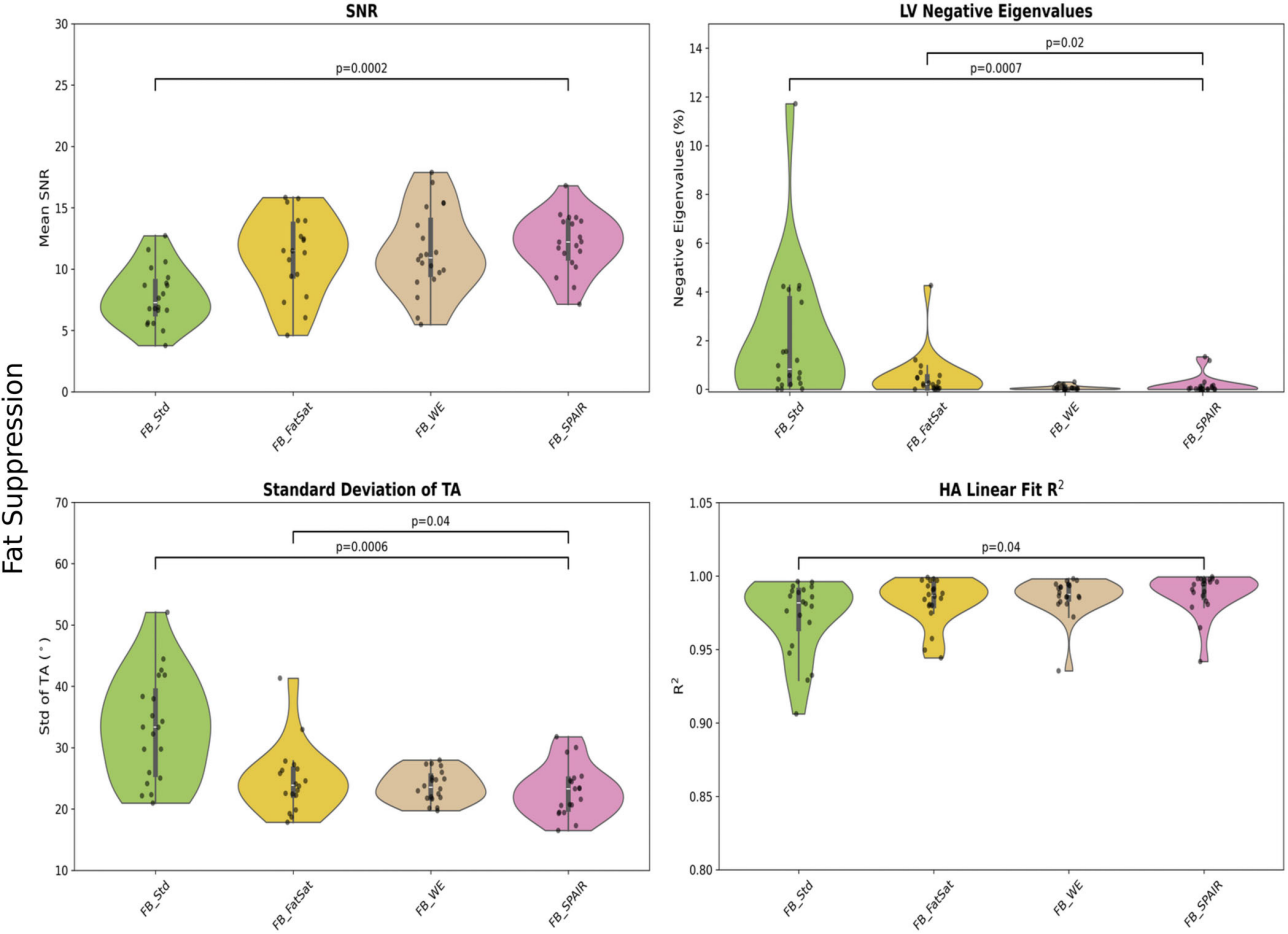
Comparison of cDTI quality metrics across fat suppression techniques including flip‐back with no suppression (standard), fat saturation, water excitation and SPAIR. Distributional violin plots for cDTI quality metrics including SNR, percentage of negative eigenvalues in the LV, the standard deviation of TA and $R^{2}$ of HA linear fit.

Comparing cDTI parameters between fat suppression techniques, Figure 7 and Figure 8 show the comparisons between the fat suppression methods using SPAIR as a reference. No suppression (0.43[0.20], p<0.01) and fat saturation (0.36[0.04], p<0.01) have higher FA values than SPAIR (0.32[0.05]). There is no significant change in MD across sequences. Absolute E2A values from fat saturation (32.7[11.8]°, p=0.02) are significantly lower than SPAIR (40.5[8.9]°). Water excitation demonstrated absolute HAG values (0.80[0.18]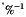) higher than SPAIR (0.74[0.18]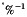), although with no significance. Absolute HAG values from no suppression (0.58[0.20]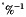) are significantly lower than SPAIR (0.74[0.18]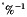, p=0.03).

Using the cDTI measures of data quality, SPAIR overall demonstrates better results than no suppression and fat saturation. SPAIR (12.2[3.0], p<0.01) achieved significantly higher SNR than no suppression (7.3[2.6]). SPAIR (0.02[0.11]%) had a significantly lower proportion of LV negative eigenvalues compared to no suppression (0.83[3.47]%, p<0.01) and fat saturation (0.21[0.45]%, p=0.02). Similarly, SPAIR (23.3[4.8]°) had lower standard deviation of TA compared to no suppression (33.3[13.5]°, p<0.01) and fat saturation (23.9[4.2]°, p=0.04), and also, SPAIR (0.994[0.012]) had higher HA $R^{2}$ compared to no suppression (0.982[0.026], p=0.04).

Water excitation and SPAIR have comparable performances. The median of absolute HAG values from water excitation (0.80[0.18]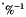) is closer to 1 than SPAIR (0.74[0.18]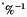). The values from standard deviation of TA also have a smaller interquartile range in water excitation (23.5[3.5]°) than SPAIR (23.3[4.8]°). However, the MD values from water excitation (1.32[0.21]$\times 10^{-3}{\mathrm{mm}}^{2}s^{-1}$) have a larger interquartile range than SPAIR (1.39[0.06]$\times 10^{-3}{\mathrm{mm}}^{2}s^{-1}$).

**FIGURE 9:**
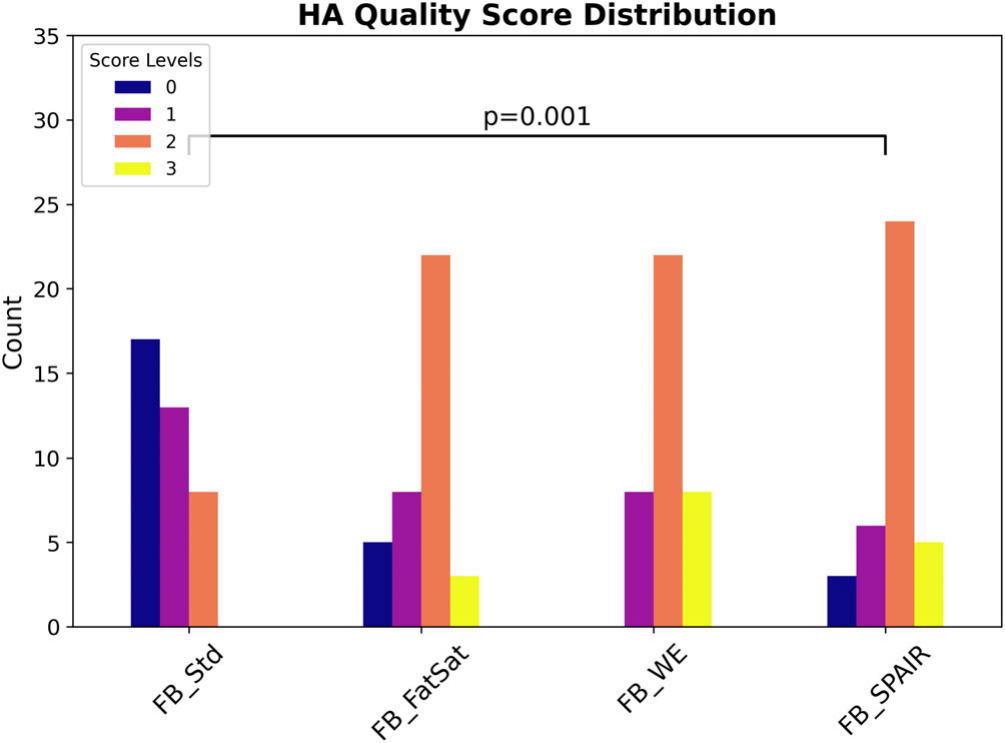
Histograms of subjective HA map quality scores for fat suppression sequences, showing the significant increases in the HA map quality between no suppression and SPAIR. Datasets scored 0 for <50% of the myocardium demonstrating the normal transmural variation in HA, scored 1 for 50%−75% normal transmural HA progression, scored 2 for 75%−95% and 3 for >95%.

**FIGURE 10:**
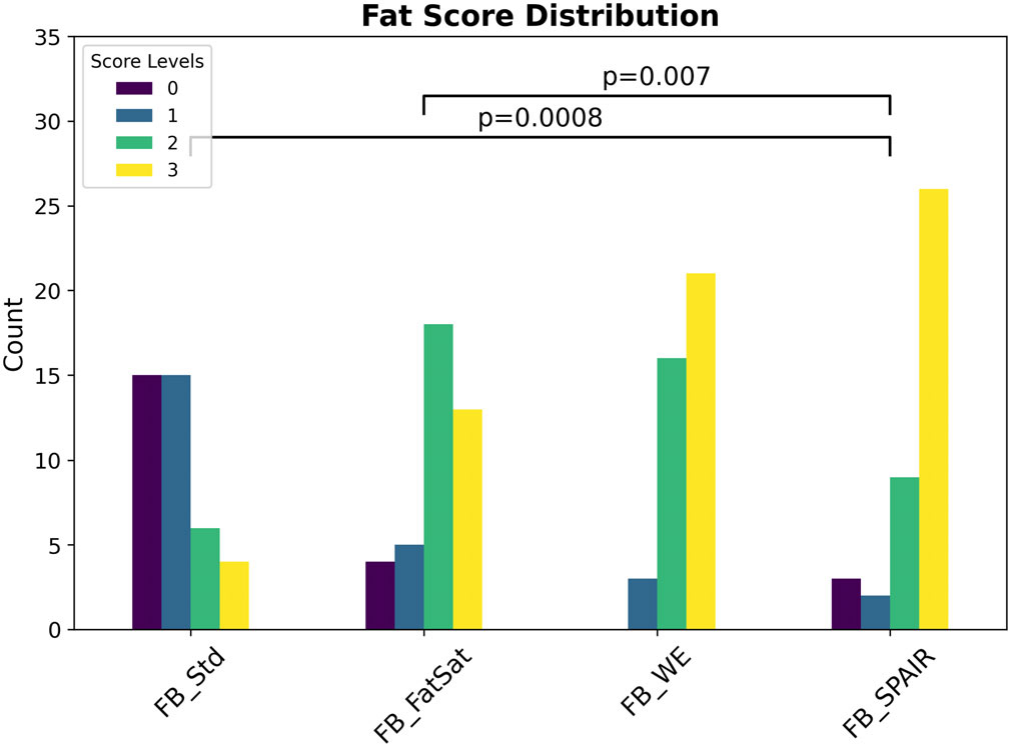
Histograms of subjective fat artifact quality scores for fat suppression sequences, showing the significant increases in the image quality scores with minimal fat artifacts from water excitation and SPAIR. Datasets scored 0 for >50% of the myocardium affected by fat artifacts, scored 1 for 25%−50% artifact appearance, scored 2 for 5%−25% and 3 for <5%.

Qualitative assessment was performed between different fat suppression techniques on HA map quality and fat artifact appearance, and results are provided in Figures 9 and 10. SPAIR (2.0[0.8]) scored higher in HA quality than no suppression (0.5[1.0], p<0.01). Moreover, SPAIR (2.8[0.6]) scored higher for the effectiveness of fat suppression than no suppression (1.0[1.5], p<0.01) and fat saturation (2.0[0.6], p<0.01). However, SPAIR does not demonstrate superior performance when compared to water excitation. The median HA quality score for water excitation was (2.0[1.0]), which is equal to SPAIR. Three of the results using SPAIR scored the lowest level, 0, for fat suppression quality, whereas no images scored 0 using water excitation. Furthermore, 93% of images acquired with water excitation scored ≥2 in fat artifact evaluation compared to 88% for SPAIR. Also, 75% of images using water excitation scored ≥2 for HA quality. These results suggest that the majority of cDTI images acquired using flip‐back with water excitation possess consistently good image quality with minimal fat artifacts.

The subjects where SPAIR fat suppression performed poorly (scored 0) are those with the highest BMI. Figure S6 demonstrates the comparison of correlation plots between fat score and BMI, for flip‐back with SPAIR and with water excitation. It can be seen that there is a negative correlation between fat score and BMI for SPAIR, with $R^{2}=0.38$ and p<0.01, but the correlation is poor for water excitation ($R^{2}\approx 0$ and p=0.96).

## 4 Discussion

We have implemented and compared reduced FOV sequences and fat suppression techniques for interleaved multislice MC‐SE cDTI. To the best of our knowledge, this is the first published work on such an optimization. We demonstrate the effectiveness of the proposed flip‐back sequence for interleaved multislice MC‐SE cDTI, and that combining this method with binomial water selective excitation or SPAIR leads to high cDTI data quality and effective fat suppression.

In the proposed flip‐back sequence, the PE FOV was reduced by applying a gradient on the $180^{{}^{\circ}}$ RF pulse in the PE direction, followed by an additional nonselective $180^{{}^{\circ}}$ flip‐back pulse to restore the inverted magnetization outside the imaged slice, thus maximizing the available signal for the acquisition of the next interleaved slice. The relatively long myocardial $T_{1}=1471\;\mathrm{ms}$ at 3 T [52] means that full longitudinal recovery after inversion or saturation requires several cardiac cycles. As a result, the PE90 and PE180 reduced FOV methods do not make efficient use of the longer TR available when interleaving slices. The flip‐back sequence design ensures each slice begins excitation with $M_{z}$ that has had a full 2 cardiac cycles to recover (assuming a two‐slice interleaved protocol as used here). The 2DRF technique avoids out‐of‐slice saturation or inversion, but has longer RF pulse durations thus leading to longer TE (TE = 65ms vs. TE = 50ms for other sequences), potentially reducing the available SNR and also has a higher specific absorption rate (SAR).

This is the first study to employ an additional nonselective pulse for reduced FOV interleaved multislice MC‐SE cDTI, with optimization for fat suppression. Similar approaches have been proposed [35] and utilized in DTI applications across cervical spinal cord [34], extraocular muscle [38] and 3D imaging [39]. Moulin et al. [53] demonstrated a similar approach for cardiac T2‐ADC mapping in a small cohort, without the ability to calculate the full diffusion tensor. However, this implementation applied the additional flip‐back pulse in the PE direction, whereas our study has shown that the use of a nonselective flip‐back pulse can increase SNR. Despite its effectiveness, a possible confound of deploying nonselective flip‐back pulse is that if the positions for basal and apical slices do not exactly align in the PE direction, then there could be signal loss along the edge of the FOV due to the $180^{{}^{\circ}}$ refocusing pulses for the two slices not acting on the same tissue.

Interleaved multislice imaging with 2DRF, demonstrated by Nguyen et al. [54], is capable of whole left ventricular coverage, and cross‐talk between slices can be minimized by deploying a reordering scheme to enforce $T_{1}$ recovery over 4 RR‐intervals. However, for breath hold applications, this approach is less effective because a high number of slices per breath hold is impractical due to the resultant long breath hold durations. Although 2DRF has a longer TE than flip‐back for the EPI readout used here, for applications that require longer EPI readouts, such as higher resolution imaging, the longer 2DRF pulse duration would be less of a disadvantage compared to flip‐back.

Based on typical myocardial $T_{2}$ values and the protocols used here, the expected signal from the flip‐back sequence would be 38% higher than from 2DRF (details are included in the Signal Ratio Calculation in Supporting Information). This theoretical difference closely aligns with our results for the $b=450\;s/{\mathrm{mm}}^{2}$ images, where we observed a 36% increase in median SNR, which was found to be significant. This observed SNR difference from flip‐back relative to 2DRF emphasizes its superiority for in vivo cDTI, given a suitable fat suppression technique.

While the PE90 sequence is not well‐suited to interleaved multislice acquisitions, the 2DRF and flip‐back sequences become more SNR efficient as TR increases, which allows for more interleaved slices. Extending breath holds beyond the 18 RR‐intervals used here is likely to be challenging, but free‐breathing approaches are a viable alternative with MC‐SE cDTI sequences [54, 55]. Interleaved two‐slice acquisition is desirable because it can increase SNR by approximately 50% due to increased $T_{1}$ recovery during the longer TR, compared to single slice imaging with TR = 1 RR‐interval. The alternative method to increase SNR is to increase the number of repetitions, assuming TR is equal to 1 RR‐interval, and doubling the number of repetitions results in increasing SNR by 41%, highlighting the high efficiency of interleaved multislice imaging. However, the gain in SNR for interleaved multislice imaging decreases as the number of interleaved slices increases. For more than four interleaved slices, the SNR gain from increasing the TR by an extra cardiac cycle and interleaving a further slice is less than 5%.

For breath hold applications, fewer directions could be acquired per breath hold in order to interleave more slices, which would also result in longer effective TR. Increasing TR is also likely to result in less variation in SNR with varying heart rate, which should improve the accuracy and precision of cDTI parameters.

Until now, the 2DRF approach might have been considered the state‐of‐the‐art for reduced FOV MC‐SE cDTI, offering effective suppression of signals outside the region of interest and compatibility with interleaved multislice imaging. However, the extended RF durations increase TE and SAR, and its specific pulse design leads to incompatibility with advanced fat suppression techniques such as binomial water selective excitation, which we have shown performs similarly to SPAIR by most measures. Furthermore, the implementation of 2DRF pulses is more complex than typical one‐dimensional slice‐selective pulses, and the 2DRF pulse option requires multichannel transmit capabilities in some vendor supplied implementations. Alternative reduced FOV strategies have been proposed including the use of saturation bands [18], although this introduces additional RF energy and we have found that the level of suppression provided by saturation bands is insufficient for reduced FOV imaging. Tilted RF excitation [56] is a sophisticated approach to reduce PE FOV whilst maintaining short TE and allowing interleaved multislice imaging, but requires complex pulse sequence design, precise slice planning and additional saturation bands. In contrast, the proposed flip‐back sequence offers an effective solution for interleaved multislice cDTI acquisition by restoring longitudinal magnetization in nonimaged slices with an additional nonselective $180^{{}^{\circ}}$ pulse. This design improves inter‐slice consistency, enhances SNR, and preserves compatibility with water excitation, making it a practical and robust alternative to more technically demanding approaches for high‐quality SE cDTI in multislice breath hold or free‐breathing protocols.

Whilst reduced FOV sequences were compared with the same fat suppression technique, namely SPAIR, a separate comparison was made with a focus on the performance of fat suppression for cDTI, utilizing the flip‐back sequence with different fat suppression techniques.

Fat saturation is simple to implement and can be flexibly embedded into different sequence designs. It requires a frequency‐selective excitation pulse to excite the fat signal, then uses an additional spoiler gradient to suppress the excited fat signal before the excitation used for imaging. However, it is sensitive to $B_{0}$ and $B_{1}$ inhomogeneities [46], which can result in failed fat suppression, and even inadvertent suppression of water signals [33]. Alternatively, SPAIR uses a frequency‐selective adiabatic pulse to accurately invert longitudinal magnetization for fat signal, even with the presence of $B_{1}$ inhomogeneity, and as fat begins to recover, imaging is initiated at TI when longitudinal magnetization for fat is zero and fat signal is nulled [44, 57]. However, SPAIR is still vulnerable to $B_{0}$ inhomogeneity, requires longer sequence duration and optimized TI. There are two options, strong and weak, for the implementation of fat saturation and SPAIR on our scanner. The difference between strong and weak options, is that strong option applies gradient reversal in the refocusing pulse, which ensures the complete removal of residual fat signals. However, in this study, weak option was chosen because gradient reversal, hence the strong option, is incompatible with the orthogonal slice‐selective gradient pairs for inner volume imaging. Both fat saturation and SPAIR involve accurate spectral selection of fat and this can be imperfect, that is, excitation and spoiling or inversion, which means residual signal in the transverse plane can occur, leading to incomplete fat suppression. Also, these methods are tuned to a particular type of fat, which may not correspond to the type of fat bordering the myocardium. In contrast, binomial water excitation does not need to selectively excite fat signal and each sub‐pulse is spatially but not spectrally selective [41, 58], and its incurred SAR level is low. However, water excitation is also sensitive to $B_{0}$ inhomogeneity [33, 46].

The significant differences observed in FA across different fat suppression techniques are likely because of lower SNR and worse performance in fat suppression. The SNR comparison in cDTI quality metrics and cDTI parameter maps for example subject with high BMI, give evidence for the significant increases in FA for no suppression and fat saturation, compared to SPAIR.

A significant increase in absolute E2A between the flip‐back sequence with fat saturation and SPAIR was observed. As supported by the significant differences in cDTI quality metrics between fat saturation and SPAIR, such as in LV negative eigenvalues and standard deviation of TA, this change in absolute E2A is likely because the images acquired with fat saturation have a lower image quality.

Binomial water excitation achieves comparable performance to SPAIR. Although it did not demonstrate superiority in cDTI quality metrics, it is the only fat suppression technique that does not have images in the lowest level for HA quality scores and fat scores. This is supported by absolute HAG values closer to 1, and the example cDTI maps of a subject with high BMI. In particular, SPAIR is likely to have lower fat suppression performance when subjects have a high BMI, whereas water excitation performs consistently regardless of the BMI level. This suggests that water excitation could be considered as a strong alternative for acquisitions in patients. However, it can be observed that for water selective excitation SNR and MD distributions have a relatively larger variability, suggesting that it is more affected by the individual differences between subjects.

SPAIR is compatible with 2DRF, and it has been shown to yield a smaller interquartile range of MD values than water excitation. However, SPAIR is sensitive to heart rate variations, and TI is critical for its performance. In this study, a fixed TI was implemented based on an assumed RR‐interval. The effectiveness of SPAIR may therefore be reduced when there is a large variability in heart rate, which is potentially more likely when scanning patients. Water excitation is less sensitive to heart rate variations. Nevertheless, the 2DRF implementation used here is incompatible with binomial excitation, because the binomial pulse is itself a composite RF pulse and the 2DRF pulses used here are too long to serve as sub‐pulses in a binomial design.

In this work, the ultra‐high gradient system enabled the implementation of sequences with very short TEs. Future work will leverage these short TEs and SNR efficiencies to boost spatial resolution beyond the relatively modest resolution used here. A potential future improvement for the flip‐back sequence is to use an adiabatic pulse for the refocusing pulse, so it can be more insensitive to field inhomogeneity.

## 5 Conclusions

The proposed flip‐back sequence effectively addresses out‐of‐slice saturation. The PE‐selective $180^{{}^{\circ}}$ refocusing pulse reduces the PE FOV, thus mitigating wrap artifacts from tissue outside the excited FOV. The additional nonselective $180^{{}^{\circ}}$ flip‐back pulse, applied immediately after the EPI readout, restores the longitudinal magnetization $M_{z}$ outside the imaged slice to closer to its maximal $M_{0}$ value, and provide more available magnetization for subsequent acquisitions in interleaved slices. This improves SNR efficiency over standard crossed‐pair orthogonal SE techniques (PE90 and PE180 here) in interleaved multislice imaging due to increased $T_{1}$ recovery. The flip‐back sequence has advantages over 2DRF pulse designs due to the shorter TE, less demanding RF requirements and compatibility with composite excitation pulses such as binomial designs. Binomial water excitation and SPAIR demonstrated superiority over other typical fat suppression methods for MC‐SE cDTI, and are both compatible with the flip‐back sequence. Binomial water selective excitation appears to have advantages in some high BMI subjects, but inter‐subject variability in some cDTI parameters is reduced with SPAIR. Flip‐back with SPAIR offers a practical approach for robust, reliable and efficient interleaved multislice cDTI with improved image quality and accuracy in cDTI parameter estimation, providing potential for clinical translation and wider application of MC‐SE cDTI.

## Funding

This work was supported by British Heart Foundation; grant: RG/F/23/110115, and EPSRC Centre for Doctoral Training in Smart Medical Imaging; co‐funded by Siemens Healthineers; grant: EP/S022104/1. Guang Yang was supported by the UKRI Future Leaders Fellowship; grant: MR/V023799/1, UKRI2738.

## Conflicts of Interest

Yaqing Luo and Ke Wen are partially funded by Siemens Healthcare. Dudley J. Pennell receives funding from Siemens. Sonia Nielles‐Vallespin reports departmental research funding from Siemens Healthcare. Andrew D. Scott reports departmental research funding from Siemens Healthcare. The other authors declare no conflicts of interest.

## Supporting information


**Figure S1:** Longitudinal magnetization diagrams for reduced FOV sequences: (A) applying 90° in both phase encoding direction (*y*) and slice selective direction (*z*), and applying 180° in *z* (2DRF); (B) applying 90° in *y*, and applying 180°in *z* (PE90); (C) applying 90°in *z*, and applying180°in *y* (PE180); (D) and finally applying 90° in *z*, applying180° in *y* with an additional nonselective 180° after the readout (flip‐back).
**Figure S2:** SNR maps for PE‐selective flip‐back sequence and nonselective flip‐back sequence from an initial phantom study.
**Figure S3:** Histograms of subjective HA map quality scores for reduced FOV sequences.
**Figure S4:** Example magnitude cDTI basal slice images to compare across subjects with normal BMI, high BMI, epicardial fat and visceral fat, to demonstrate the effectiveness of flip‐back with water excitation and with SPAIR.
**Figure S5:** Comparison of example cDTI maps at both slices for the flip‐back sequence with different fat suppression techniques, for an example subject with high BMI.
**Figure S6:** Comparison of correlation plots between fat score and BMI, for flip‐back with SPAIR and with water excitation respectively.

## Data Availability

The data that support the findings of this study are available from the corresponding author upon reasonable request. Post‐processing was performed using the open‐source Python pipeline “INDI” developed by our group https://github.com/ImperialCollegeLondon/INDI [47, 48].
