# Supplementary material for: Optimized Reduced Field of View and Fat Suppression Methods for Interleaved Multislice In Vivo Cardiac Diffusion Tensor Imaging
Source: Magn Reson Med. 2026 Apr 22;96(3):1097–110. doi: 10.1002/mrm.70394 (PMC13327497; doi:10.1002/mrm.70394)
Supplement: Supplementary file 1 — Figure S1: Longitudinal magnetization diagrams for reduced FOV sequences: (A) applying 90° in both phase encoding direction (y) and slice selective direction (z), and applying 180° in z (2DRF); (B) applying 90° in y, and applying 180°in z (PE90); (C) applying 90°in z, and applying180°in y (PE180); (D) and finally applying 90° in z, applying180° in y with an additional nonselective 180° after the readout (flip‐back). Figure S2: SNR maps for PE‐selective flip‐back sequence and nonselective flip‐back sequence from an initial phantom study. Figure S3: Histograms of subjective HA map quality scores for reduced FOV sequences. Figure S4: Example magnitude cDTI basal slice images to compare across subjects with normal BMI, high BMI, epicardial fat and visceral fat, to demonstrate the effectiveness of flip‐back with water excitation and with SPAIR. Figure S5: Comparison of example cDTI maps at both slices for the flip‐back sequence with different fat suppression techniques, for an example subject with high BMI. Figure S6: Comparison of correlation plots between fat score and BMI, for flip‐back with SPAIR and with water excitation respectively. [file MRM-96-1097-s001.pdf]

SUPPORTING INFORMATION

The following supporting information is available as part of the online article:

Figure S1. Longitudinal magnetisation diagrams for reduced FOV sequences: (A) applying 90° in both phase encoding direction (y) and slice selective direction (z), and applying 180° in z (2DRF); (B) applying 90° in y, and applying 180° in z (PE90); (C) applying 90° in z, and applying 180° in y (PE180); (D) and finally applying 90° in z, applying 180° in y with an additional non-selective 180° after the readout (Flip-back).

Figure S2. SNR maps for PE-selective flip-back sequence and non-selective flip-back sequence from initial phantom study.

Figure S3. Histograms of subjective HA map quality scores for reduced FOV sequences.

Figure S4. Example magnitude cDTI basal slice images to compare across subjects with normal BMI, high BMI, epicardial fat and visceral fat, to demonstrate the effectiveness of flip-back with water excitation and with SPAIR.

Figure S5. Comparison of example cDTI maps at both slices for the flip-back sequence with different fat suppression techniques, for an example subject with high BMI.

Figure S6. Comparison of correlation plots between fat score and BMI, for flip-back with SPAIR and with water excitation respectively.

Signal Ratio Calculation From theoretical modelling, it can be calculated that assuming TI=TR-TE/2≈TR, and the signal model that considers both T_1 recovery and T_2 decay, $S = M_0 \cdot \left(1 - e^{-TR/T_1}\right) \cdot e^{-TE/T_2}$, the signal ratio between flip-back and 2DRF is 1.38, meaning that given $T_1 = 1471ms$ and $T_2 = 47ms$ for myocardium[?] , 2DRF has TE $= 65ms$ and TR = 2 RR-intervals, and flip-back has TE $= 50ms$ and TR = 2 RR-intervals, the expected signal from flip-back is 38% higher in this protocol setting.

□

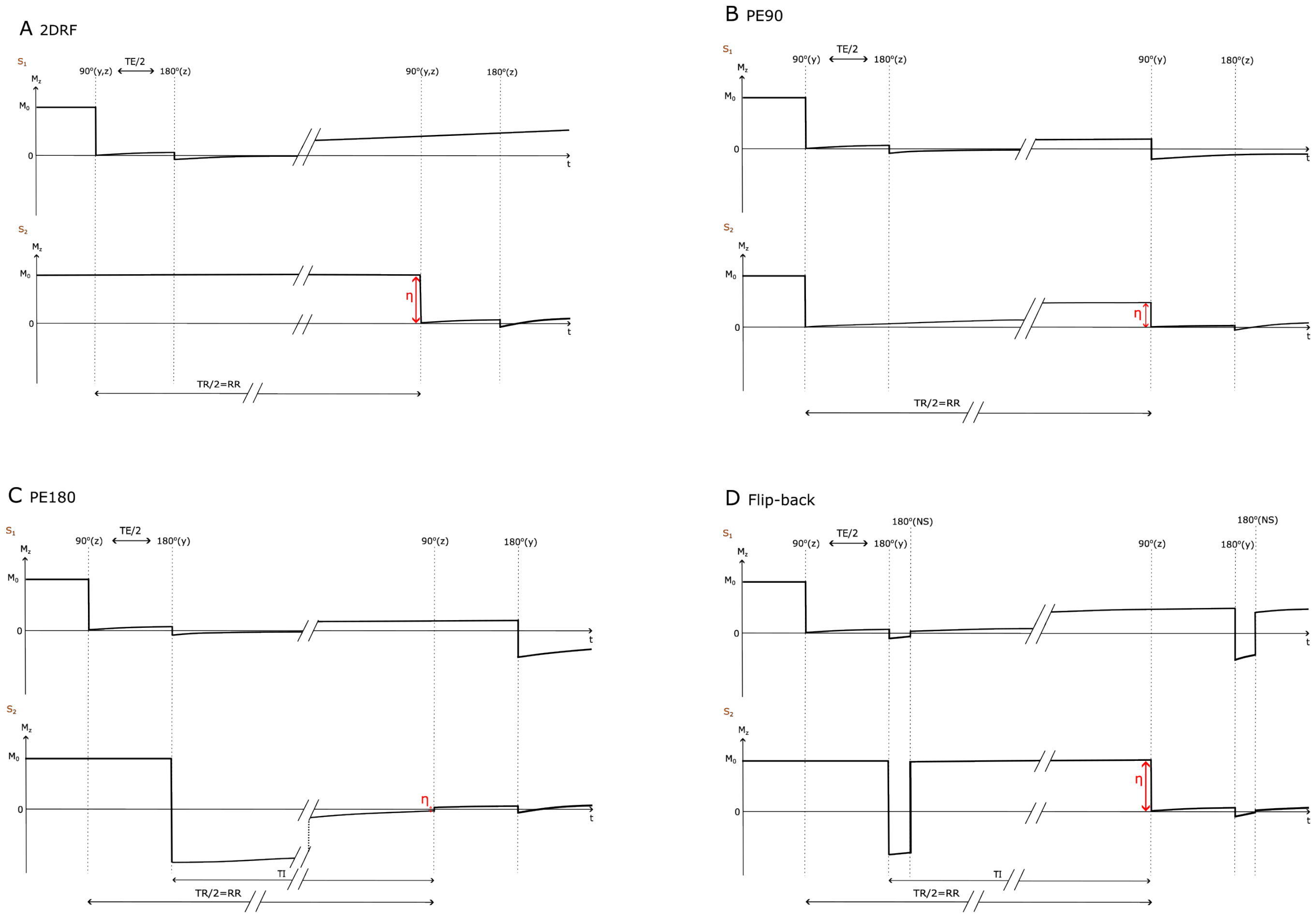

Figure S1. Longitudinal magnetisation diagrams for slice 1 (S1) and slice 2 (S2) acquisitions in interleaved two-slice reduced FOV sequences: (A) applying 90° in both phase encoding direction (y) and slice selective direction (z), and applying 180° in z (2DRF); (B) applying 90° in y, and applying 180° in z (PE90); (C) applying 90° in z, and applying 180° in y (PE180); (D) and finally applying 90° in z, applying 180° in y with an additional non-selective (NS) 180° after the readout (Flip-back). η represents the amount of longitudinal magnetisation available to tip into the transverse plane and hence the signal available for imaging.

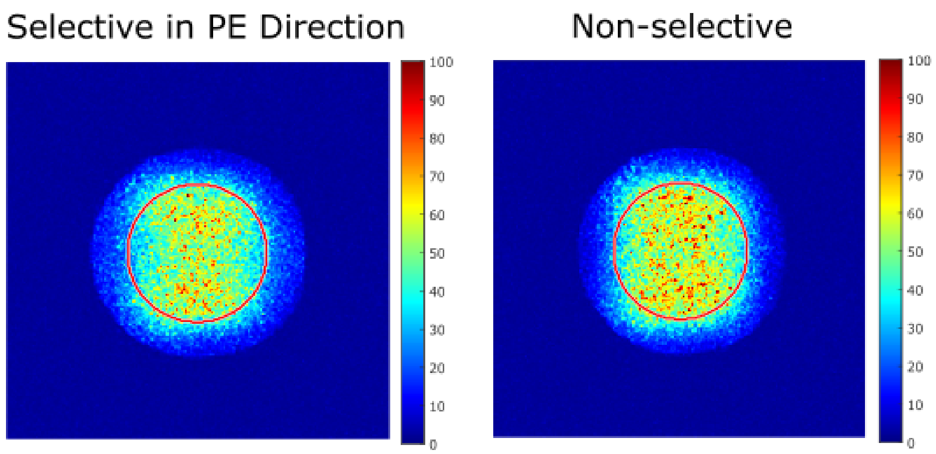

Figure S2. Comparison SNR maps for applying a PE-selective flip-back pulse and non-selective flip-back pulse. The first applies 90° pulse in SS direction, then 180° in PE direction, followed by another 180° in the PE direction. The second case applies excitation and refocusing pulses in the same directions, but with a non-selective 180° flip-back pulse. For each case, 20 repetitions of $b = 0s/mm^2$ images were acquired. The mean and standard deviation for the SNR values inside the selected ROI outlined by the red circles, are 49.0(12.5) and 54.2(11.8) respectively for PE-selective and non-selective flip-back pulses. This was applied on agar phantom (3 litres including water, 120g of agar power), $T_1 = 1270ms$, $T_2 = 38ms$, with diameter of around 196mm.

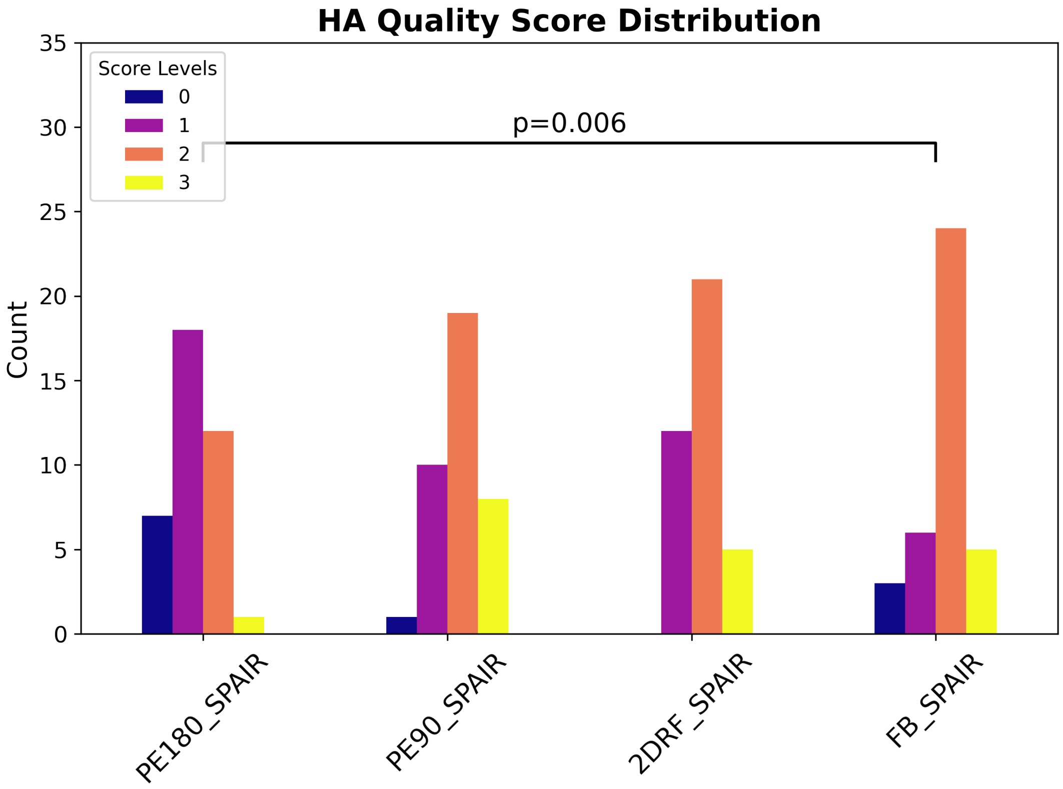

Figure S3. Histograms of subjective HA map quality scores for reduced FOV sequences. Datasets scored 0 for $< 50\%$ of the myocardium demonstrating the normal transmural variation in HA, 1 for $50 - 75\%$ normal transmural HA progression, 2 for $75 - 95\%$ and 3 for $> 95\%$.

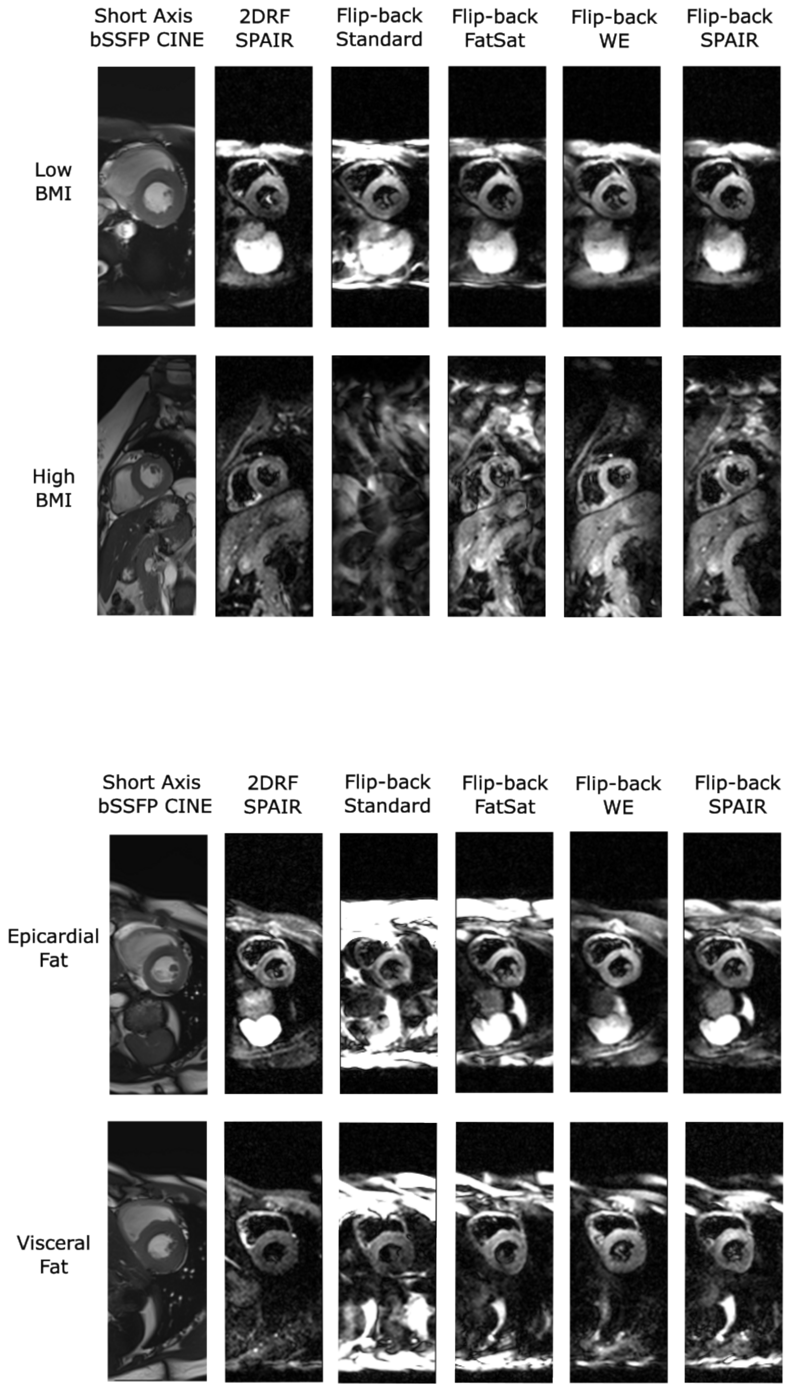

Figure S4. Example magnitude cDTI basal slice images for subjects with normal BMI, high BMI, epicardial fat and visceral fat. Results from flip-back with water excitation and SPAIR demonstrate the best image quality, with no visible artefacts.

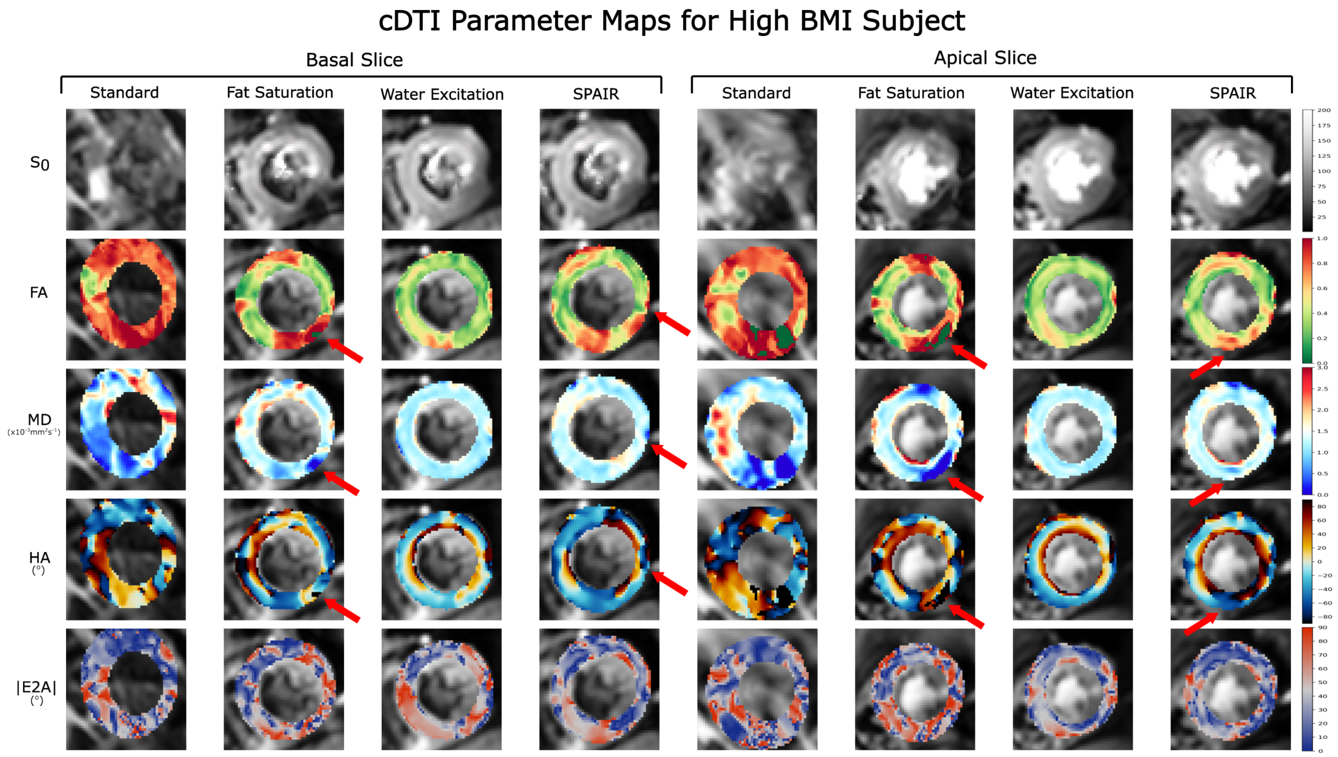

Figure S5. Comparison of example cDTI maps including average magnitude image, FA, MD, HA and absolute E2A, at basal and apical slices for fat suppression techniques with the flip-back sequence including no suppression (standard), fat saturation, water excitation and SPAIR, for an example subject with high BMI. Red arrows highlight some example regions affected by fat artefacts. No fat suppression (standard) failed in this case. Results from the flip-back sequence with binomial water excitation are the least affected by fat artefacts.

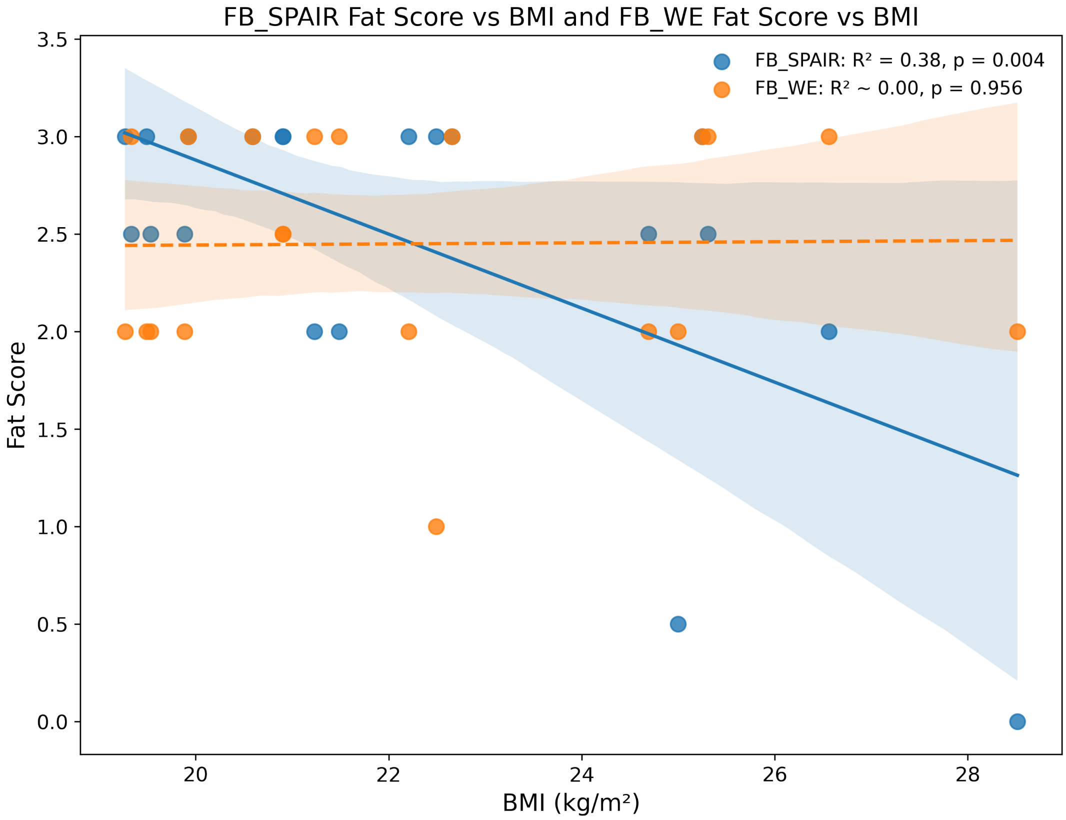

Figure S6. Comparison of correlation plots between fat score and BMI, for flip-back with SPAIR and with water excitation respectively. Each point on the figure represents the median value between the fat scores for basal slice and apical slice. For SPAIR, there is a significant negative correlation between fat score and BMI, meaning that subjects with higher BMI are more likely to have lower image quality, but the correlation is negligible for water excitation.
